# Research in eating disorders: the misunderstanding of supposing serious mental illnesses as a niche specialty

**DOI:** 10.1007/s40519-022-01473-9

**Published:** 2022-09-09

**Authors:** Enrica Marzola, Matteo Panero, Paola Longo, Matteo Martini, Fernando Fernàndez-Aranda, Walter H. Kaye, Giovanni Abbate-Daga

**Affiliations:** 1 Eating Disorders Center, Department of Neuroscience, University of Turin, Turin, Italy; 2 Department of Psychiatry, Bellvitge University Hospital, Barcelona, Spain; 3 CIBER Fisiopatologia Obesidad Y Nutrición (CIBERobn), Instituto de Salud Carlos III, Barcelona, Spain; 4 Department of Clinical Sciences, School of Medicine and Health Sciences, University of Barcelona, Barcelona, Spain; 5 Psychoneurobiology of Eating and Addictive Behaviors Group, Neurosciences Programme, Bellvitge Biomedical Research Institute (IDIBELL), 08908 Barcelona, Spain; 6 Department of Psychiatry, University of California, San Diego, San Diego, CA USA

**Keywords:** Anorexia nervosa, Bulimia nervosa, Binge eating disorder, Schizophrenia, Bibliometry, Bradford’s law

## Abstract

**Purpose:**

Eating disorders (EDs) are mental illnesses with severe consequences and high mortality rates. Notwithstanding, EDs are considered a niche specialty making it often difficult for researchers to publish in high-impact journals. Subsequently, research on EDs receives less funding than other fields of psychiatry potentially slowing treatment progress. This study aimed to compare research vitality between EDs and schizophrenia focusing on: number and type of publications; top-cited articles; geographical distribution of top-ten publishing countries; journal distribution of scientific production as measured by bibliometric analysis; funded research and collaborations.

**Methods:**

We used the Scopus database, then we adopted the Bibliometrix R-package software with the web interface app Biblioshiny. We included in the analyses 1,916 papers on EDs and 6491 on schizophrenia.

**Results:**

The ED field published three times less than schizophrenia in top-ranking journals – with letters and notes particularly lacking—notwithstanding a comparable number of papers published per author. Only 50% of top-cited articles focused on EDs and a smaller pool of journals available for ED research (i.e., Zones 1 and 2 according to Bradford's law) emerged; journals publishing on EDs showed an overall lower rank compared to the schizophrenia field. Schizophrenia research was more geographically distributed and more funded; in contrast, a comparable collaboration index was found between the fields.

**Conclusion:**

These data show that research on EDs is currently marginalized and top-rank journals are seldom achievable by researchers in EDs. Such difficulties in research dissemination entail potentially serious repercussions on clinical advancements.

**Level of evidence:**

Level V: opinions of respected authorities, based on descriptive studies, narrative reviews, clinical experience, or reports of expert committees.

**Supplementary Information:**

The online version contains supplementary material available at 10.1007/s40519-022-01473-9.

## Introduction

Almost 20 years have passed since Frost and colleagues [1] published their research on the possible bias against eating disorders (EDs), considered trivial and self-inflicted among some leading psychiatric journals. Since then, bewildering advances have been made in the light of EDs as biologically driven illnesses [2], although stigmatization is still present and a real burden for many patients [3]. Noteworthy, part of this “blame” could be within the ED field itself. Several clinicians and researchers start their careers with the idea that EDs are caused by psychosocial factors and are substantially a socially learned behavior [4]. That is, they tend to lack interest or understanding of how genetics and neurobiology contribute to EDs. This is despite limited evidence that treatments based on psychosocial factors or learned behaviors have much efficacy [5]. In fact, EDs are plagued by countless organic and psychological sequelae and have among the highest mortality rates of mental disorders [6, 7]. Only 50% of patients respond to state-of-the-art treatments and about 20% develop an enduring ED [8]. A multidisciplinary approach is strictly needed, which is costly and hard to find for many patients [9]. Also, differently from other fields of psychiatry, the pharmacological armamentarium available for patients with EDs is at best scarce. With that said, it is noteworthy that annually EDs cost the US more than $65 billion [10] and the UK 6.8–8 billion GBP [11]. When hospitalization is needed, data from Canada showed that about 36,000 EUR are required as overall costs for society [12]; notwithstanding, up to 40% of patients need to be readmitted within 12 months after discharge [13].

Within this background, it should be also considered that research funding, quality, and availability often resonate with patients’ standard of care. The publication system plays a role in this scenario as well since, for example, the number of citations is used by universities and funding bodies as a proxy for scientific outcomes [14]. Citations are highly valued also for grant applications by which researchers with a strong track record in high-impact and widely cited journals are likely to become the funding recipients. Without being able to publish in high-impact journals, research can be difficultly disseminated at the expense of patients. In fact, it has been recently authoritatively stated: "the loss of academic programs has rendered treatment for AN a luxury commodity" [15], and "no formal training structures are in place for young researchers in Europe that reflect the transdisciplinary needs of the EDs specialty" [16]. This is particularly true in the field of EDs, since patients require a highly specialized multidisciplinary approach, with individualized treatment plans targeting each motivation phase. As a result, only a minority of patients with EDs receive proper care (report UK), and frequently after a prolonged (and costly) untreated illness [17]. Taken together, these data show how compelling is the need for extensive research on evidence-based treatments [18], notwithstanding inadequate funding in both Europe [16, 19] and the USA [15].

Therefore, with the overarching aim to clarify the extent of current research productivity and visibility in the ED field from a top-rank journal perspective, we decided to perform a comparison of publications in top-rank psychiatry journals between EDs and schizophrenia. This rationale was grounded on some key points that guided our research: first, EDs represent biologically based serious mental illnesses deserving health care coverage and overall research/funding/clinical attention comparable to conditions currently categorized as “severe mental illnesses”, including schizophrenia [20]. Second, when looking at the disability-adjusted life years (DALYs), EDs and schizophrenia showed the closest DALYs across psychiatric disorders (179 for EDs versus 262 for schizophrenia) and overall similar prevalence worldwide [21] although EDs are even more common in western countries [22]. Third, despite the scant research available on both diagnoses, the family burden has been found as comparable, mostly concerning supervision needed [23]. Fourth, also the quality of life between patients with EDs and schizophrenia was reported as similar [24, 25]. Finally, some treatment-related difficulties, like relapse prevention strategies [26], non-responders, and the frequent need for hospital readmissions [13, 27], are widely shared characteristics between the fields. Notwithstanding such similarities, it should be noted that EDs are plagued by increasing years lived with disabilities when compared to schizophrenia [21].

With more detail, this study aimed to measure the total number of scientific papers published between 2018 and 2020 in the top-ranking journals according to the Scopus database with respect to categories of Psychiatry and Mental Health comparing the ED and schizophrenia fields. In particular, for both fields, we measured and compared: (a) number and type of publications; (b) top-cited articles; (c) the geographical distribution of top-ten publishing countries; (d) analysis of bibliometric indices measuring journal and author distribution of scientific production; (e) the extent of funded research and collaborations. We expected to find a reduced overall scientific production focused on EDs concerning all the aforementioned endpoints.

## Materials and methods

### Disability-adjusted life-years for eating disorders and schizophrenia

Following earlier research [1], we used disability-adjusted life-years (DALY) to find a suitable psychiatric diagnosis to compare to EDs. DALYs are a measure of the burden of a certain disease taking into account the degree of illness, disability, and long-term survival. DALYs derive from the sum of the years of life lost due to premature mortality and the years lived with a disability thus one DALY is conceived as one lost year of “healthy” life. We referred to the latest estimates of the Global Burden of Disease available here https://vizhub.healthdata.org/gbd-compare/. It should be borne in mind when reading the analyses that DALYs for EDs are likely to be underestimated: in fact, the Global Burden of Disease has not included so far two major ED diagnoses, namely Binge Eating Disorder and Other Specified Eating Disorder, thus receiving criticisms in this regard [28], also in the light of Binge Eating Disorder being the most prevalent ED [29]. With that being said, we referred to the 15–49 age range for Western Europe reporting the following DALYs per 100,000 [30]: 179 for EDs, 646 for anxiety disorders, 262 for schizophrenia, 748 for major depression, 270 for bipolar disorders. Therefore, schizophrenia was set as a comparison diagnosis since it resulted to be the closest disease regards to DALYs.

### Setting/variables

The literature search was conducted in the Scopus database between July and October 2021. We selected the Scopus database because is one of the largest and most inclusive databases [31], and provides for every journal the CiteScore, an index specifically designed for bibliometric analyses.

The CiteScore of a journal is the number of citations received in that year and the previous 3 years for documents published in the journal during that period, divided by the total number of published documents in the journal during the previous three-year period. Furthermore, the Scopus database allows the selection of journals pertaining to definite Subject Areas. With these instruments, we searched for papers published in a three-year time span (2018–2019–2020), in journals that resulted in the first (highest) quartile based on 2019 CiteScore Index in the subject area of Psychiatry and Mental Health. We retrieved 166 journals (sources) which were in the first quartile in accordance with the 2019 CiteScore.

The rationale for relying on this three-year time span is to have a broad and up-to-date area of research: the number of citations may vary after publication so a three-year span may offer a good balance, relying on citations referring to a reasonably long period. Importantly, setting Citescore 2019 as a year for the choice of the first quartile to identify the most important journals means that this index is not affected by the rise of SARS-COV-2 whose impact on scientific publications and journals’ bibliometric indexes remains to be determined, and could have otherwise biased the results of our analysis (e.g., with the inclusion of journals which have had a steep increase in citations due to the specific focus on highly cited COVID-19 papers in 2020) [32, 33].

Nonetheless, the scientific production of 2020, even though partly affected by the pandemic, was included in our analysis in order to provide comprehensive data on this triennium.

For EDs and schizophrenia we used Medical Subject Headings (MeSH) Terms and MeSH Tree Structure to determine a broad and at the same time precise bibliometric research. As a subsequent step, we developed two query strings (fully available in the Supplementary Materials): the first aimed to search articles in the field of EDs (using the following Mesh terms: ("eating disorder" OR "eating disorders" OR bulim* OR anorex* OR binge OR purging OR arfid OR "feeding disorder" OR "feeding disorders" OR "other specified feeding" OR pica OR purging OR "night eating" OR ( rumination AND ( eating OR food))), the second in that of schizophrenia (using the following Mesh terms: ( schizophren*) OR (psychosi* AND NOT ( Dementia* OR Bipolar OR “affective disorder” OR Substance OR Capgras OR Parasitosis))). Four authors (E. M., M. P., P. L., M. M.) conducted an independent search on Scopus focused on both strings to exclude non-eligible items; then, working in couples, two teams of researchers compared the retrieved papers and, in case of disagreement, discussed and clarified eventual conflicts; in case of need, the senior author (G. A. D.) was contacted to resolve the discordance.

Papers were included if they were about studies either (1) including samples of individuals diagnosed with the disorder of interest or (2) explicitly designed to investigate illness-related constructs. The query string on EDs retrieved a total of 2915 papers and, after double-check selection 1916 papers were retained, with a discordance rate of 11.6%. The query string on schizophrenia yielded 7688 papers of which, after item-by-item selection, 6,491 were retained, with a discordance rate of 10.8%. (see also Supplementary Fig. 1: Prisma Diagram).

All metadata from articles were downloaded with information for all fields, including the author, affiliation, title, source, language, document type, keywords, indexed-Keywords, fundings, abstract, and references. These data sources were used for the bibliometric analysis.

### Data sources/measurement

Data acquired from the Scopus collection, as mentioned above, were exported in Bibtex format and then used. For this study, we used the Bibliometrix R-package software developed by Aria and Cuccurullo [34] and written in the R language. The Bibliometrix R-package software is open-source and provides a set of instruments to conduct quantitative research in bibliometrics. Moreover, the web interface app called Biblioshiny for Bibliometrix supports scholars in easy use of the main features of Bibliometrix and has been used in this study to import data from Scopus in BibTex format.

This was not a human-subject study; therefore, neither approval by the institutional review board nor obtaining informed consent was required.

### Bibliometric tools

Bradford law is one of the most used bibliometrics tools. Samuel Bradford described the scattered distribution of bibliometric references [35]. In a specific area of interest, when dividing references into three zones of equal numerosity, the citations for the first zone would come from a small "core" group of journals. The second zone requires more journals to achieve the same number of citations, and the third zone exponentially more than the second. In moving from Zone 1 to Zone 3 journals have a "diminishing productivity" which has become known as Bradford's law of scattering (that is also presented as "1:n:n^2^") or Bradford's distribution. It follows that to reach a complete bibliographic coverage for a specific topic, it is necessary to analyze an exponentially growing number of peripheric journals, albeit a small group of core journals encloses the highest number of references.

## Results

### Comparison of published research over 2018–2020 in the top-ranking journals for the eating disorders and schizophrenia fields

As shown in Table 1, 1916 and 6491 papers on EDs and schizophrenia, respectively, were published over the triennium 2018–2020, according to Scopus. Average years from the publication were overall comparable, as well as the average citations per document and the average citations per year per document.

**Table 1:** Scientific publications in journals 1st quartile and authors according to Scopus from 2018 to 2020 for the eating disorders and schizophrenia fields

Triennium 2018–2020	EDs	Schizophrenia
	*n* (n/tot documents)	*n* (*n*/tot documents)
Journals	113	140
Documents	1916	6491
Average years from publication	1.83	1.99
Average citations per document	8.02	8.4
Average citations per year per doc	2.69	2.65
Authors	6740 (3.51)	23,141 (3.56)
Author appearances	11,341(5.91)	52,151(8.03)
Authors of single-authored documents	57 (0.03)	307 (0.05)
Authors of multi-authored documents	6683 (3.48)	22,834 (3.51)

Similar to the data on papers, also authors in the ED field were much fewer than those publishing on schizophrenia. Author appearances were greater for schizophrenia, as well as the authors of single-authored documents.

When considering the type of article, as shown in Table 2, the ED field showed an imbalance mostly concerning letters and notes when compared to schizophrenia: letters and notes in schizophrenia are published more and with higher percentages (8.8% and 2.7% vs 1.6% and 1.2%, respectively). Annual paper distribution is described in Table 1 in Supplementary Materials.

**Table 2:** Type of articles published in the triennium 2018–2020 according to Scopus for the eating disorders and schizophrenia fields

	EDs	Schizophrenia
	*n* (%)	*n* (%)
*Type of document*		
Article	1604 (83.8)	4854 (74.9)
Editorial	40 (2.1)	178 (2.8)
Review	215 (11.2)	633 (9.8)
Letter	31 (1.6)	573 (8.8)
Note	24 (1.2)	180 (2.7)
Conference paper	2 (0.1)	17 (0.2)
Short survey	–	30 (0.4)
Others	–	26 (0.4)

Moreover, out of a total of 166 journals ranked in the first quartile according to Scopus (year 2019 which was chosen as the year to select quartiles for journals for the Psychiatry and Mental Health category), 113 (68%) published papers on EDs and 140 (84%) published papers on schizophrenia. With respect to highly specialized journals, four could be retrieved for the ED field: International Journal of Eating Disorders (rank = 57), European Eating Disorders Review (rank = 67), Eating and Weight Disorders (rank = 121), and Eating Behaviors (rank = 128) and three (showing an overall higher rank) for schizophrenia: Schizophrenia Bulletin (rank = 13) NPJ Schizophrenia (rank = 38) and Schizophrenia Research (rank = 58).

Table 3 shows the most relevant journals for both fields (see also Supplementary Figs. 2 and 3), according to the Biblioshiny tool. Only three journals overlapped between the fields (i.e., Psychological Medicine, BMC Psychiatry, and Journal of Psychiatric Research).

**Table 3:** Most relevant journals of the triennium 2018 – 2020 according to Scopus for the eating disorders and schizophrenia fields

Eating disorders			Schizophrenia		
Journal	Rank	*n* of documents	Journal	Rank	*n* of documents
International journal of eating disorders	57	518	Schizophrenia research	58	1523
Eating and weight disorders	121	328	Schizophrenia bulletin	13	457
European eating disorders review	67	178	Early intervention in psychiatry	127	281
Eating behaviors	128	131	Psychological medicine	19	276
Psychological medicine	19	49	Translational psychiatry	23	229
Journal of adolescent health	48	34	BMC psychiatry	103	179
Journal of affective disorders	52	29	Journal of psychiatric research	46	162
Journal of psychiatric research	46	25	Molecular psychiatry	5	158
BMC psychiatry	103	24	Journal of clinical psychopharmacology	129	141
Current opinion in psychiatry	51	23	European archives of psychiatry and clinical neuroscience	69	134

### Top-cited articles for the eating disorders and schizophrenia fields

When the ten top-cited articles were analyzed (see Table 4), 50% of most cited papers on EDs were not focused on EDs but rather included an ED sample while investigating a broader or transdiagnostic psychiatric aspect (e.g., comorbidity, dissociation). Therefore, five papers were focused on EDs (of which two were on EDs and SARS-COV-2) although it was included one paper on orthorexia, currently not a formal ED diagnosis. In contrast, 8 out of 10 papers in the schizophrenia field were strictly focused on schizophrenia; that is, the vast majority reported “schizophrenia” or “psychotic disorders” or “psychosis” in the title. Also, when considering the rank of the journals of the top-cited publications, the highest rank was 19 in the ED field (i.e., Psychological Medicine) while, schizophrenia-focused papers were published in top-rank journals, including World Psychiatry (rank = 1). See Table 4 for all details.

**Table 4:** Ten top-cited articles (TC = total citations) in the triennium 2018 – 2020 according to Scopus for the eating disorders and schizophrenia fields

Eating disorders					Schizophrenia				
Paper	Journal rank	TC	TC per year	Normalized TC	Paper	Journal rank	TC	TC per year	Normalized TC
Huang Y, et al. Prevalence of mental disorders in China: a cross-sectional epidemiological study. Lancet Psychiatry. 2019 PMID: 30,792,114	3	344	114.7	38.1	Huang Y, et al. Prevalence of mental disorders in China: a cross-sectional epidemiological study. Lancet Psychiatry. 2019 PMID: 30,792,114	3	344	114.7	40.8
Plana-Ripoll O, et al. Exploring Comorbidity Within Mental Disorders Among a Danish National Population. JAMA Vinood PatelPsychiatry. 2019 PMID: 30,649,197; PMCID	4	126	41	13.6	Kelly S, et al. Widespread white matter microstructural differences in schizophrenia across 4322 individuals: results from the ENIGMA Schizophrenia DTI Working Group. Mol Psychiatry. 2018 PMID: 29,038,599	5	221	55.2	16.9
Lyssenko L, et al. A Meta-Analysis of Studies Using the Dissociative Experiences Scale. Am J Psychiatry. 2018 PMID: 28,946,763	6	103	25.7	8.3	Mcguire et al. Cannabidiol (CBD) as an Adjunctive Therapy in Schizophrenia: A Multicenter Randomized Controlled Trial. Am J Psychiatry. 2018 PMID: 29,241,357	6	214	53.5	16.4
Fernández-Aranda F, et al. COVID-19 and implications for eating disorders. Eur Eat Disord Rev. 2020 PMID: 32,346,977	67	95	47.5	20.8	Di Forti M, et al. The contribution of cannabis use to variation in the incidence of psychotic disorder across Europe (EU-GEI): a multicenter case–control study. Lancet Psychiatry. 2019 PMID: 30,902,669	3	207	69	24.5
Cena H, et al. Definition and diagnostic criteria for orthorexia nervosa: a narrative review of the literature. Eat Weight Disord. 2019 PMID: 30,414,078	121	94	31.3	10.4	Charlson FJ, et al. Global Epidemiology and Burden of Schizophrenia: Findings From the Global Burden of Disease Study 2016. Schizophr Bull. 2018 PMID: 29,762,765	13	202	50.5	15.5
Udo T, Grilo CM. Psychiatric and medical correlates of DSM-5 eating disorders in a nationally representative sample of adults in the United States. Int J Eat Disord. 2019 PMID: 30,756,422	57	83	27.7	9.2	Correll CU, et al. Comparison of early intervention services vs treatment as usual for early-phase psychosis: a systematic review, Meta-analysis, and meta-regression. JAMA Psychiatry. 2018 PMID: 29,800,949	4	200	50	15.4
India State-Level Disease Burden Initiative Mental Disorders Collaborators. The burden of mental disorders across the states of India: the Global Burden of Disease Study 1990–2017. Lancet Psychiatry. 2020 PMID: 31,879,245	3	78	39	17.1	Radua J, et al. What causes psychosis? An umbrella review of risk and protective factors. World Psychiatry. 2018 PMID: 29,352,556	1	184	46	14.1
Phillipou A, et al. Eating and exercise behaviors in eating disorders and the general population during the COVID-19 pandemic in Australia: Initial results from the COLLATE project. Int J Eat Disord. 2020 PMID: 32,476,163	57	78	39	17.1	Lai MC, et al. Prevalence of co-occurring mental health diagnoses in the autism population: a systematic review and meta-analysis. Lancet Psychiatry. 2019 PMID: 31,447,415	3	175	58.3	20.7
Thompson PM et al. ENIGMA Consortium. ENIGMA and global neuroscience: A decade of large-scale studies of the brain in health and disease across more than 40 countries. Transl Psychiatry. 2020 PMID: 32,198,361	23	78	39	17.1	Duncan LE et al. Largest GWAS of PTSD (N = 20 070) yields genetic overlap with schizophrenia and sex differences in heritability. Mol Psychiatry. 2018 PMID: 28,439,101	5	168	42	12.9
Brockmeyer T, et al. Advances in the treatment of anorexia nervosa: a review of established and emerging interventions. Psychol Med. 2018 PMID: 28,889,819	19	65	16.2	5.2	Rehm J, Shield KD. Global Burden of Disease and the Impact of Mental and Addictive Disorders. Curr Psychiatry Rep. 2019 PMID: 30,729,322	44	153	51	18.1

### Geographical distribution of the ten-top publishing countries for the eating disorders and schizophrenia fields

As shown in Table 5, concerning the world distribution of the 10 most productive countries, Asian countries were present for schizophrenia while both China and Japan were absent (as well as other Asian countries) for EDs publications. The USA, Europe, and Oceania were particularly represented in both research areas.

**Table 5:** Ten top-publishing countries in the triennium 2018 – 2020 according to Scopus for the eating disorders and schizophrenia fields

EDs	Number of documents (2018–2020)	Schizophrenia	Number of documents (2018–2020)
USA	2740	USA	8063
Germany	618	UK	3281
UK	604	China	2735
Australia	584	Australia	2087
Italy	474	Germany	1838
Canada	387	Canada	1826
Spain	273	Spain	1601
France	267	France	1319
Sweden	241	Japan	1298
Netherlands	211	Netherlands	1235

### Journal distribution of the scientific production

According to Bradford's law [35], when all the references in a certain field are equally divided into three zones, the citations for Zone 1 would come from a small core group of journals. Zone 2 would require more journals to achieve the same number of citations, and Zone 3 exponentially more than the second. Therefore, from Zone 1 to Zone 3 there is a decrease in productivity.

Interestingly, as reported in Table 6, the ED field reported fewer journals in both Bradford Law’s Zones 1 (greatest productivity) and 2 (moderate productivity); with more detail, the difference was particularly marked in Zone 2, with 6 journals (5,3%) available for EDs and 15 (10,7%) for schizophrenia. That is, researchers in the ED field have a reduced pool of highly cited journals available to consider when submitting their research.

**Table 6:** Productivity-based top journals for the eating disorders and schizophrenia fields

Eating disorders		Schizophrenia	
Journal	Rank (*n*. of documents)	Journal	Rank (*n*. of documents)
Zone 1 = great productivity			
International journal of eating disorders	57 (518)	Schizophrenia research	58 (1523)
Eating and weight disorders	121 (328)	Schizophrenia bulletin	13 (457)
		Early intervention in psychiatry	127 (281)
Zone 2 = moderate productivity			
1.European eating disorders review	67 (178)	1. Psychological medicine	19 (276)
2. Eating behaviors	128 (131)	2. Translational psychiatry	23 (229)
3. Psychological medicine	19 (49)	3. BMC Psychiatry	103 (179)
4. Journal of adolescent health	48 (34)	4. Journal of psychiatric research	46 (162)
5. Journal of affective disorders	52 (29)	5. Molecular psychiatry	5 (158)
6. Journal of psychiatric research	46 (25)	6. Journal of clinical psychopharmacology	129 (141)
		7. European archives of psychiatry and clinical neuroscience	69 (134)
		8. Jama psychiatry	4 (126)
		9. American journal of psychiatry	6 (117)
		10. Australian and New Zealand journal of psychiatry	47 (104)
		11. European neuropsychopharmacology	36 (100)
		12. Psychiatry research—neuroimaging	133 (99)
		13. The lancet psychiatry	3 (97)
		14. Neuropsychopharmacology	12 (96)
		15. Psychiatric services	130 (95)
Zone 3 = low productivity			
Eating disorders *n* = 105 journals (92.9%)		Schizophrenia *n* = 122 journals (87.1%)	

Also, when considering the ranks of Zone 2 journals, the schizophrenia field could rely on high-impact journals. The highest-impact journal for the ED field in Zone 2 (*n* = 6; 5,3%) was Psychological Medicine (rank = 19) while, in the schizophrenia field (*n* = 15; 10,7%), 5 journals reported an even higher rank (i.e., Lancet Psychiatry, rank = 3; JAMA Psychiatry, rank = 4; Molecular Psychiatry, rank = 5; American Journal of Psychiatry, rank = 6; Neuropsychopharmacology, rank = 12; see Table 6).

Concerning Zone 3, namely the one including less relevant journals to the field, it is noteworthy that the vast majority of journals (*n* = 105, 92.9%) in the ED field was in this zone while a smaller proportion of journals were in Zone 3 for schizophrenia (*n* = 122, 87.1%).

### Funding and collaborations in the eating disorders and schizophrenia fields

With respect to funded research, only 51.9% (996/1916) of papers on EDs acknowledged being funded while 78.1% (5075/6491) of papers in the field of schizophrenia did.

Concerning collaborations, documents per author and collaboration index were overall comparable between fields (i.e., 3.6 for EDs and 3.75 for schizophrenia, see Figs. 4, 5, 6, 7 in Supplementary Materials, and Table 7) although overall fewer co-authors per document were present in ED publications.

**Table 7:** Collaboration data over the 2018–2020 triennium according to Scopus for the eating disorders and schizophrenia fields

Triennium 2018–2020	EDs	Schizophrenia
Documents per author	0.284	0.28
Authors per document	3.52	3.57
Co-authors per documents	5.92	8.03
Collaboration index	3.6	3.75

## Discussion

With this paper we aimed to measure research productivity about EDs over the triennium 2018–2020, comparing the ED and the schizophrenia fields. Five main findings emerged: first, the schizophrenia field published three times as much as the EDs in top-ranking journals; second, the vast majority of top-cited papers on schizophrenia were strictly focused on psychosis-related topics, while 50% of top-cited papers on EDs only incidentally included EDs while investigating a broader psychiatric construct (i.e., dissociation); also, in contrast with ED research, schizophrenia-focused top-cited papers were frequently published in top-rank journals. Third, no Asian countries were included in the top-ten publishing countries on EDs while both China and Japan resulted included in the list of schizophrenia top publishing countries. Fourth, when compared to the schizophrenia field, ED research resulted to have much fewer candidate journals able to assure a decent number of citations, also characterized by a much lower rank. Finally, research published on EDs was less funded than that of the counterpart but collaborations were overall similar.

Interestingly, the two research fields, yielded a much diverse number of publications over the same triennium: in fact, over 6000 papers were available on schizophrenia while less than 2000 for EDs, even despite an overall comparable burden of illness (i.e., DALYs; GBD 2017 Disease and Injury Incidence and Prevalence Collaborators, 2018; see material and methods) and a growing incidence for EDs [36]. Moreover, data showed that researchers on EDs represent a small community but equally productive when compared to that of schizophrenia: in fact, the number of authors and documents have similar proportions (i.e., EDs:schizophrenia = 1:3) between research areas. Notwithstanding this difference in raw data, it is noteworthy that the number of citations per year per document was overall comparable thus highlighting the interest of the readership for EDs. However, it is noteworthy that letters and notes were particularly lacking for EDs. These kinds of papers seldom offer novel data but rather propose comments, experts' opinions, and perspectives, or novel ideas needing further investigation thus representing a proxy for the vitality of the scientific debate around a certain topic. As previously suggested [37], editorials are usually invited and represent an overall contribution to the increase of the citation count of articles in a certain journal. Therefore, both quantity and quality of the scientific production – published in the first quartile of top-rank journals—have been found as much diverse between fields. This is of interest since it has been consistently reported how the impact factor and the prestige of the publishing journal can be the most important predictor of future citations [38] and how it can be relevant to researchers' careers and funding [39]. Although some papers reported an acceleration in rates of scientific publications on EDs [40, 41] we emphasize that the access of research on EDs to top-rank journals is still a much different issue.

In line with the considerations on the author's career and funding possibilities, when considering the top-cited articles over the 2018–2020 triennium, eight out of ten papers on schizophrenia were focused on this diagnosis while, for EDs, 50% of top-cited papers included a sample of patients with EDs but investigated other psychiatric constructs. Also, on average, the ED-focused papers were published in journals with a much more modest impact factor when compared to that of the counterpart. Taken together, these data highlight how research dissemination and citation can be difficult for EDs. Also, this finding somehow downsizes the comparability of the number of citations per year per document; in fact, when top-cited papers are considered, the divergence between fields tends to confirm how EDs are seen as a niche specialty [19]. Also, 2 out of 5 top-cited ED-focused papers dealt with SARS-COV-2-related aspects; in fact, EDs widely grew during the SARS-COV-2 pandemic [42], highlighting the crucial relevance of such diagnoses in this area, especially in young people.

Another parameter concerned the world distribution of the ten top-publishing countries. Schizophrenia research showed a balanced distribution of the most productive countries, with all continents involved. In contrast, in the ED field, Asian countries resulted to be not represented despite the increasing trend of ED incidence in Asian countries which has been recently reported, mostly in China, also given its rapid economic growth [43]. The contribution of Asian countries to the ED research field would be much needed since the prevalence of EDs among female Chinese university students was found to be similar to that reported in Western countries [44]; however, it has been reported that only two specialized centers for the treatment of EDs are currently available in China thus potentially making difficult for patients being engaged in treatment and research studies [45]. However, as the study of EDs is still in its infancy in non-Western countries, it is possible that many publications in other languages are not always available in the online libraries used and are not published in journals in the first quartile. Nevertheless, this date underlines how research in schizophrenia is certainly more widespread worldwide.

It is commonly experienced by researchers who work in the ED field the paucity of suitable candidate journals for scientific papers [19]. In a substantial number of submissions, even before starting the peer-review process, the Editorial Board let the authors know that the topic is not listed in the aims of the journal, has low priority, or does not fit the needs of the readership. Therefore, frequently it is advised to consider more specialized journals. Another relevant issue is that there are special considerations for ED research, such as the impact of nutrition on pathophysiology and that journals or study sections need reviews by experts who take this into consideration. Overall, our findings support this everyday experience since, when considering data from Bradford Law analysis, journals in Zone 1, namely including the journals that are the most frequently cited in the literature of the field eventually sparking the highest interest in the readership, two are available for EDs, and three for schizophrenia. In this regard, more important data came from the analysis of the so-called Zone 2. In fact, journals in Zone 2 represent the pool of journals that can ensure decent citations when the specialized/most productive ones on a certain topic (i.e., Zone 1) are excluded. Therefore, in the ED field, the pool of Zone 2 included only 6 (5,3%) journals while 15 (10,7%) were available for schizophrenia. Moreover, when reading these data, it should be also borne in mind that all journals in Zone 1 for schizophrenia had a higher rank than those included in Zone 1 for EDs. Similarly, Zone 2 for schizophrenia included top-rank journals (i.e., the Lancet Psychiatry, JAMA Psychiatry, Molecular Psychiatry, the American Journal of Psychiatry, Neuropsychopharmacology) which appear only in Zone 3 in the ED field. Although the Bradford law over the years has been criticized [46], it should be noted that the aim of this paper was not to perform a strict bibliometric analysis of scientific production but rather to explore and measure what could play a role in making research productivity and visibility so difficult in the ED field. With that being said, this is something that could be far from being specific for the ED field. For example, it has been reported how also literature on anxiety disorders, notwithstanding great prevalence and huge DALYs, represents only 4–7% of manuscripts in high impact psychiatry journals [37].

Finally, research published on EDs was less funded than its counterpart. Although mental health tends to receive little funding attention at a broader level [47, 48], this tends to become particularly true for EDs. For example, the European Research Council, a major European funding institution for cutting-edge research, when listing psychiatric disorders states: LS5_12 Psychiatric disorders (e.g. schizophrenia, autism, Tourette's syndrome, obsessive–compulsive disorder, depression, bipolar disorder, attention deficit hyperactivity disorder) [49]. This is just an example of a lack of visibility that could ultimately affect patients' treatment. Recent data from Australia showed that schizophrenia received funding 60 times higher than those allocated to EDs [50]. Funding is particularly welcome in the ED field to implement formal training structures able to provide the extensive multidisciplinary skills that are required to work with patients with EDs whose clinical needs are particularly complex [15, 16]. Still, in contrast with the ED field, schizophrenia research can rely on multiple pharmacological strategies which may have specific funding agencies in pharmaceutical industries, further boosting research productivity.

In contrast, scientific collaborations were comparable between fields, showing that both communities put a great deal of effort into implementing their networks thus developing researchers’ expertise and enhancing data collection and a greater flow of information, ultimately increasing funding chances [51].

In closing, this paper explored and measured research productivity and vitality in the ED field also by comparing it to that of schizophrenia. Despite some strengths including the use of straightforward criteria and methods ensuring replicability of the findings, some limitations should be acknowledged as well: first, we a priori selected certain parameters including the use of DALYs, triennium time span, having only one comparator (schizophrenia), database (i.e., Scopus), and database-related categories (i.e., Scopus Psychiatry and Mental Health). As a result, relevant journals and highly cited papers may have not been included according to the aforementioned parameters for both fields (i.e., Nature genetics: Watson et al., Genome-wide association study identifies eight risk loci and implicates metabo-psychiatric origins for anorexia nervosa, 2019; Lam M et al. Comparative genetic architectures of schizophrenia in East Asian and European populations, 2019). In future research it may be useful to use other parameters, such as those used by the web of science system (WOS) or a longer period, for example 5 years and more Scopus categories. Also, we reiterate that DALYs currently do not include relevant ED diagnoses so the data on EDs may be underestimated. Furthermore, in our analysis we couldn’t calculate the total number of researchers in a field and if their number differs among the two fields. Finally, funding data present some possible biases as they could be underrepresented as not necessarily all authors acknowledge their funding in their papers or may have multiple fundings available at the time, and the number (quantity) of funded papers may not represent the total amount of funding (quality) in a field. That said, these data suggest that research on EDs is currently limited in getting ED-focused papers published in top-rank journals, obtaining funding, and conveying an adequate geographic representation. This lack of breadth and vitality of scientific production could ultimately end in slow advancements in treatments.

Strength and limits: the study evaluates the quality and amount of research in the ED and schizophrenia field using straightforward criteria and methods, ensuring replicability of the findings. The main limit is the a priori selection of specific parameters including the use of DALYs, triennium, database, and database-related categories.

What is already known on this subject: EDs are severe mental illnesses with high mortality rates, and significant DALYs, but for researcher is difficult to publish on EDs in high-impact journals. Subsequently, research on EDs receives less funding than other fields of psychiatry and it is known that research funding, quality, and availability influence patients’ standard of care.

What this study adds? Bibliometric data offer a fine grained overview of a research field. Findings show that the ED field, compared to schizophrenia, published much less and in journals with an overall lower rank. Moreover, research on EDs is less funded and worldwide distributed. These data highlight the difficulty in dissemination and citation for the ED field that could be reflected in fewer funding opportunities leading thus to a potential slowing in treatment progress.

## Supplementary Information

Below is the link to the electronic supplementary material.

Supplementary file1 (DOCX 570 KB)

## Data Availability

The data that support the findings are available from the corresponding author upon reasonable request.
