# Supplementary material for: Research in eating disorders: the misunderstanding of supposing serious mental illnesses as a niche specialty
Source: Eat Weight Disord. 2022 Sep 9;27(8):3005–16. doi: 10.1007/s40519-022-01473-9 (PMC9462607; doi:10.1007/s40519-022-01473-9)
Supplement: Supplementary file 1 — Supplementary file1 (DOCX 570 KB) [file 40519_2022_1473_MOESM1_ESM.docx]

**Supplementary Materials**

**S1. Query strings to search articles in the field of eating disorders.**

SRCID ( 6200180159 OR  4600151524 OR   21100356804  OR 21100228547 OR 17382 OR 17664 OR 3600148102 OR 12854 OR 19400158464 OR 16757 OR 19181 OR 18017 OR 19353 OR 15402 OR 24745 OR 17643 OR 14364 OR 15370 OR 18742 OR 19700177555 OR 24749 OR 13487 OR 19900191866 OR 17574 OR 15440 OR 22452 OR 14990 OR 15439 OR 20106 OR 21100403234 OR 13502 OR 14313 OR 15452 OR 18745 OR 12127 OR 15576 OR 16685 OR 21100854140 OR 19700182728 OR 15375 OR 16816 OR 13442 OR 14838 OR 15011 OR 21100373947 OR 16812 OR 14250 OR 15357 OR 145178 OR 21100363813 OR 14994 OR 16245 OR 19900192160 OR 23109 OR 19172 OR 28534 OR 12675 OR 19355 OR 27473 OR 15548 OR 14277 OR 130029 OR 94948 OR 21100415705 OR 10600153347 OR 11700154316 OR 14097 OR 21100884825 OR 15546 OR 16246 OR 15300154855 OR 26380 OR 14799 OR 21100461914 OR 19841 OR 12841 OR 15369 OR 24791 OR 15578 OR 16203 OR 30042 OR 25372 OR 19700175164 OR 18594 OR 15665 OR 26613 OR 16072 OR 18712 OR 15582 OR 24763 OR 19489 OR 16664 OR 25334 OR 14921 OR 13491 OR 17545 OR 25229 OR 29164 OR 13069 OR 14016 OR 21100894555 OR 14260 OR 21100228093 OR 16672 OR 7000153203 OR 16200 OR 30045 OR 15313 OR 16817 OR 24451 OR 25655 OR 16188 OR 17500155127 OR 19560 OR 17554 OR 29451 OR 28478 OR 12053 OR 15029 OR 15460 OR 21100394256 OR 20814 OR 16102 OR 16249 OR 17639 OR 17500155156 OR 13545 OR 23008 OR 110175 OR 14077 OR 26411 OR 18730 OR 12070 OR 19885 OR 14872 OR 5700155920 OR 18701 OR 25231 OR 12020 OR 12000154320 OR 21100881252 OR 70201 OR 21100899528 OR 29471 OR 27628 OR 12004 OR 19700166518 OR 25291 OR 19173 OR 16705 OR 19700167014 OR 16726 OR 12054 OR 4000151904 OR 16400154776 OR 23164 OR 36956 OR 17539 OR 15626 OR 17400154828 OR 21100203112 OR 12123 OR 30408 OR 19900191617 OR 21100801744 OR 15584

)  AND  ( PUBYEAR  =  2020  OR  PUBYEAR  =  2019  OR  PUBYEAR  =  2018  )  AND  TITLE-ABS-KEY ( "eating disorder"  OR  "eating disorders"  OR  bulim*  OR   anorex*  OR  binge  OR  purging   OR  arfid  OR  "feeding disorder"  OR  "feeding disorders"  OR  "other specified feeding"  OR   pica  OR  purging  OR   "night eating"  OR   ( rumination  AND  ( eating  OR  food ) ) )

**S2. Query strings to search articles in the field of schizophrenia.**

SRCID ( 6200180159 OR  4600151524 OR   21100356804  OR 21100228547 OR 17382 OR 17664 OR 3600148102 OR 12854 OR 19400158464 OR 16757 OR 19181 OR 18017 OR 19353 OR 15402 OR 24745 OR 17643 OR 14364 OR 15370 OR 18742 OR 19700177555 OR 24749 OR 13487 OR 19900191866 OR 17574 OR 15440 OR 22452 OR 14990 OR 15439 OR 20106 OR 21100403234 OR 13502 OR 14313 OR 15452 OR 18745 OR 12127 OR 15576 OR 16685 OR 21100854140 OR 19700182728 OR 15375 OR 16816 OR 13442 OR 14838 OR 15011 OR 21100373947 OR 16812 OR 14250 OR 15357 OR 145178 OR 21100363813 OR 14994 OR 16245 OR 19900192160 OR 23109 OR 19172 OR 28534 OR 12675 OR 19355 OR 27473 OR 15548 OR 14277 OR 130029 OR 94948 OR 21100415705 OR 10600153347 OR 11700154316 OR 14097 OR 21100884825 OR 15546 OR 16246 OR 15300154855 OR 26380 OR 14799 OR 21100461914 OR 19841 OR 12841 OR 15369 OR 24791 OR 15578 OR 16203 OR 30042 OR 25372 OR 19700175164 OR 18594 OR 15665 OR 26613 OR 16072 OR 18712 OR 15582 OR 24763 OR 19489 OR 16664 OR 25334 OR 14921 OR 13491 OR 17545 OR 25229 OR 29164 OR 13069 OR 14016 OR 21100894555 OR 14260 OR 21100228093 OR 16672 OR 7000153203 OR 16200 OR 30045 OR 15313 OR 16817 OR 24451 OR 25655 OR 16188 OR 17500155127 OR 19560 OR 17554 OR 29451 OR 28478 OR 12053 OR 15029 OR 15460 OR 21100394256 OR 20814 OR 16102 OR 16249 OR 17639 OR 17500155156 OR 13545 OR 23008 OR 110175 OR 14077 OR 26411 OR 18730 OR 12070 OR 19885 OR 14872 OR 5700155920 OR 18701 OR 25231 OR 12020 OR 12000154320 OR 21100881252 OR 70201 OR 21100899528 OR 29471 OR 27628 OR 12004 OR 19700166518 OR 25291 OR 19173 OR 16705 OR 19700167014 OR 16726 OR 12054 OR 4000151904 OR 16400154776 OR 23164 OR 36956 OR 17539 OR 15626 OR 17400154828 OR 21100203112 OR 12123 OR 30408 OR 19900191617 OR 21100801744 OR 15584

)  AND  ( PUBYEAR  =  2020  OR  PUBYEAR  =  2019  OR  PUBYEAR  =  2018  )  AND  TITLE-ABS-KEY  ( schizophren*) OR (psychosi*  AND NOT ( Dementia*  OR  Bipolar OR “affective disorder” OR Substance OR Capgras OR Parasitosis ) )

**Supplementary Table 1 - Annual Paper Distribution**

| Year | Documents | |
| --- | --- | --- |
|  | ED | Schizophrenia |
| 2018 | 548 | 2260 |
| 2019 | 571 | 1956 |
| 2020 | 796 | 2272 |

**Supplementary Figure 1. Prisma diagrams
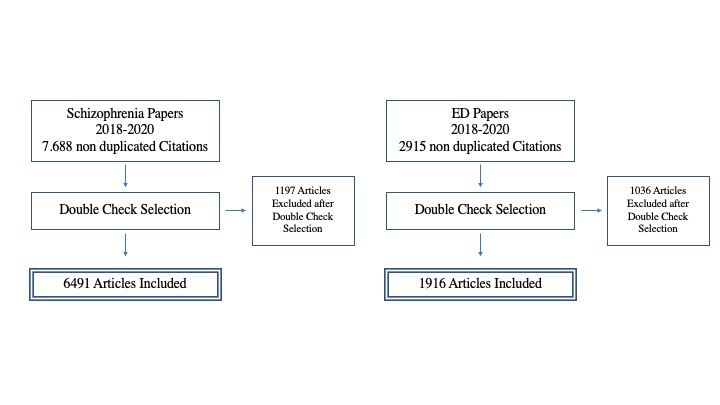
**

**Supplementary Figure 2. Ten most relevant sources for eating disorders research in the triennium 2018-2020 according to Scopus.**


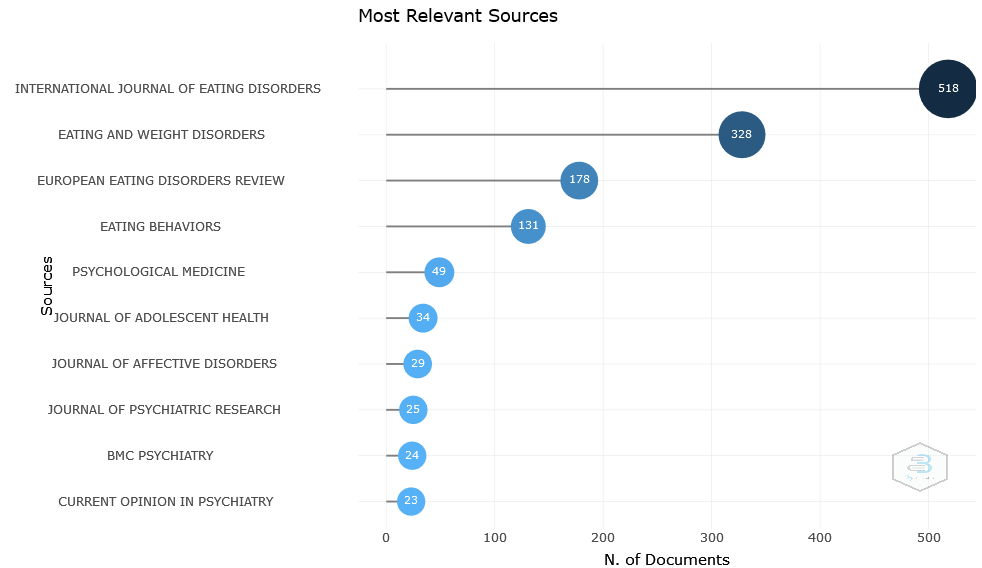


**Supplementary Figure 3. Ten most relevant sources for schizophrenia research in the triennium 2018-2020 according to Scopus.**

**
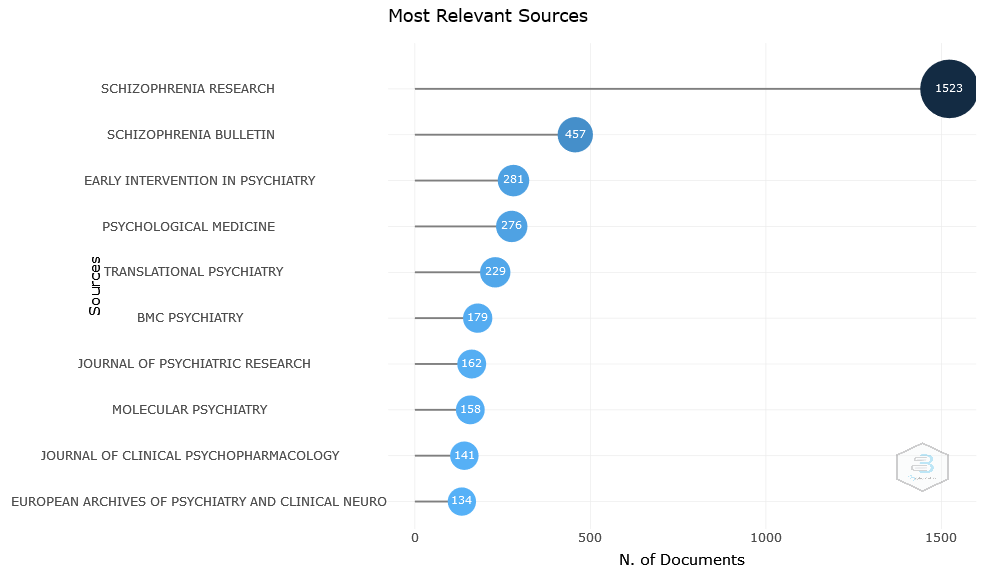
**

**Supplementary Figure 4. Clusters of authors’ collaborations of research on eating disorders in the triennium 2018-2020 according to Scopus.**

**
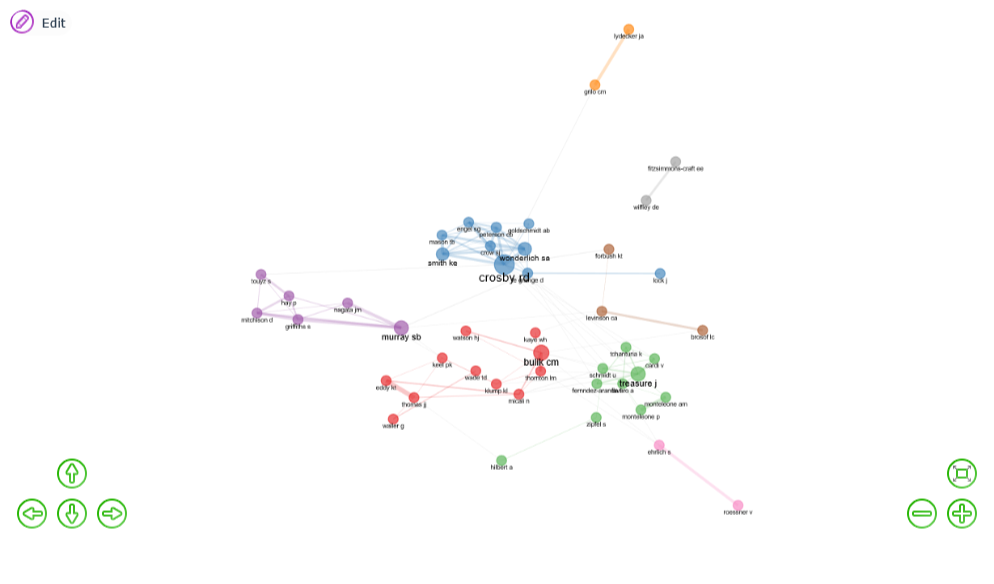
**

**Supplementary Figure 5. Clusters of authors’ collaborations of research on schizophrenia in the triennium 2018-2020 according to Scopus.**


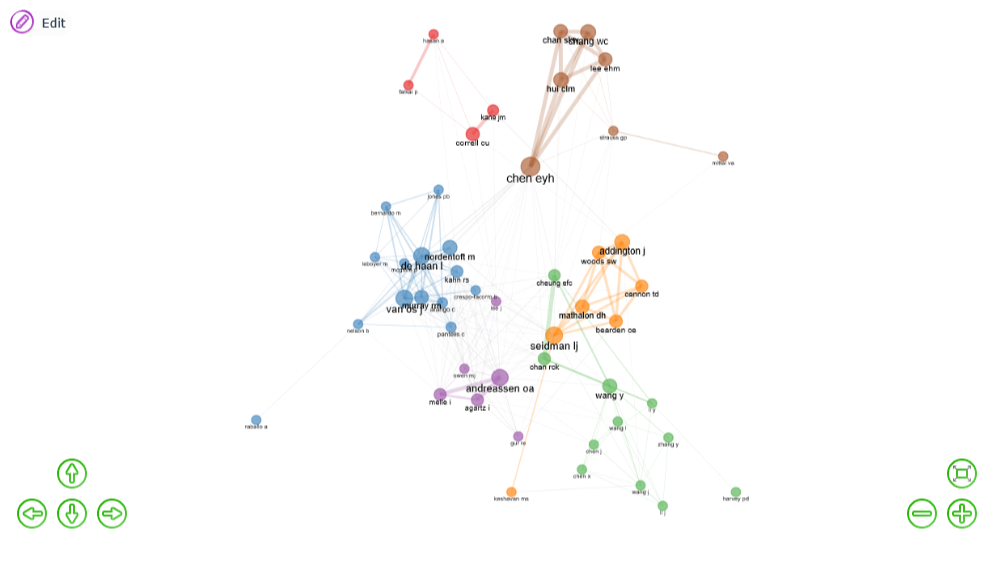


**Supplementary Figure 6. Worldwide distribution of collaborations for research on eating disorders in the triennium 2018-2020 according to Scopus.**

**
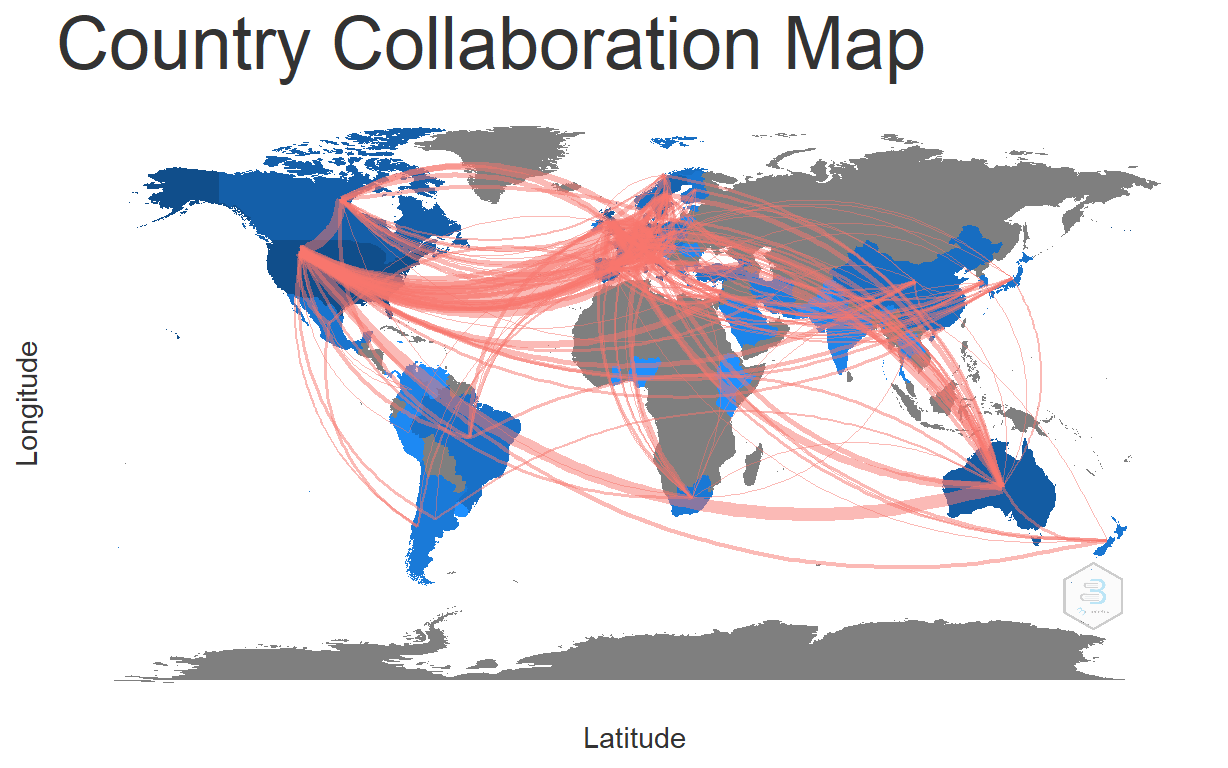
**

**Supplementary Figure 7. Worldwide distribution of collaborations for research on schizophrenia in the triennium 2018-2020 according to Scopus.**

**
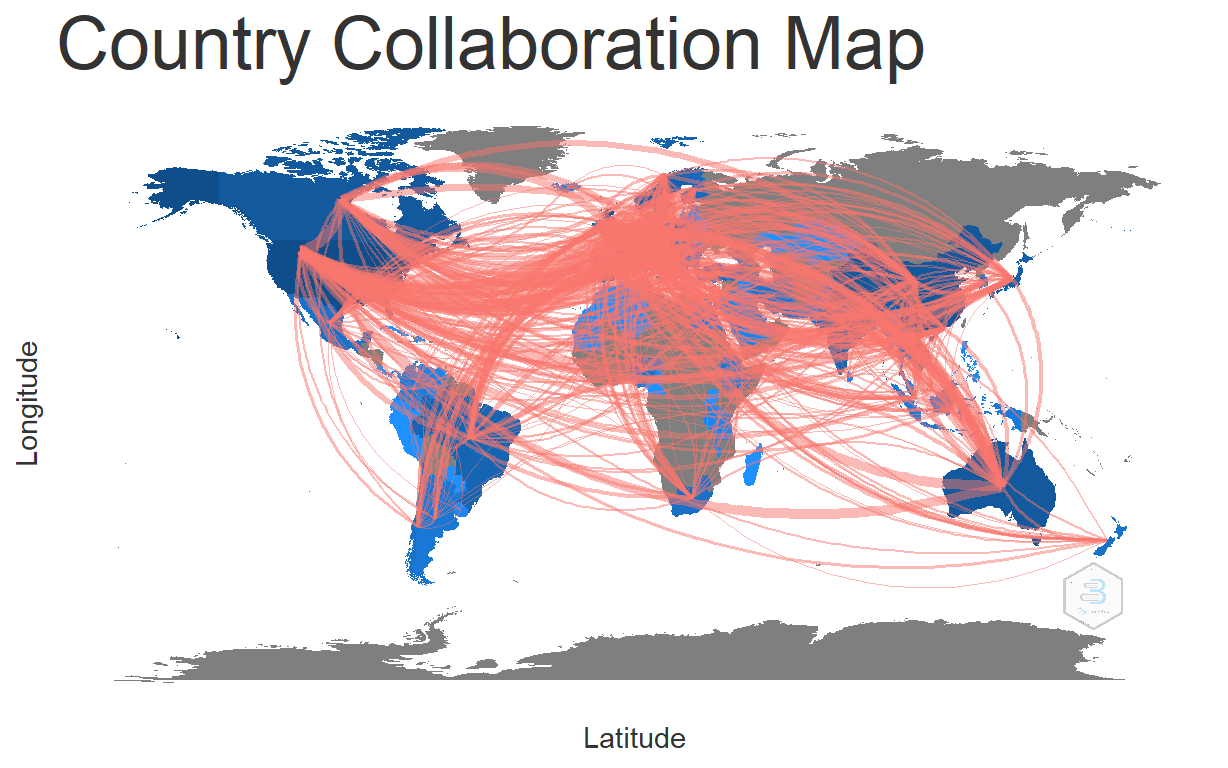
**
